# Peritoneal fibrosis: from pathophysiological mechanism to medicine

**DOI:** 10.3389/fphys.2024.1438952

**Published:** 2024-09-04

**Authors:** Yingxi Kang, Yuan Liu, Ping Fu, Liang Ma

**Affiliations:** ^1^ Department of Nephrology, Institute of Kidney Diseases, West China Hospital of Sichuan University, Chengdu, China; ^2^ Health Management Center, General Practice Medical Center, West China Hospital, Sichuan University, Chengdu, China

**Keywords:** peritoneal fibrosis, pathophysiology, therapeutics, peritoneal dialysis, mechanism

## Abstract

Peritoneal dialysis (PD) is currently one of the effective methods for treating end-stage renal disease (ESRD). However, long-term exposure to high concentration glucose in peritoneal dialysis environment could lead to peritoneal fibrosis (PF), impaired peritoneal filtration function, decreased peritoneal dialysis efficiency, and even withdrawal from peritoneal dialysis in patients. Considerable evidence suggests that peritoneal fibrosis after peritoneal dialysis is related to crucial factors such as mesothelial-to-mesenchymal transition (MMT), inflammatory response, and angiogenesis, etc. In our review, we summarize the pathophysiological mechanisms and further illustrate the future strategies against PF.

## Introduction

**FIGURE 1:**
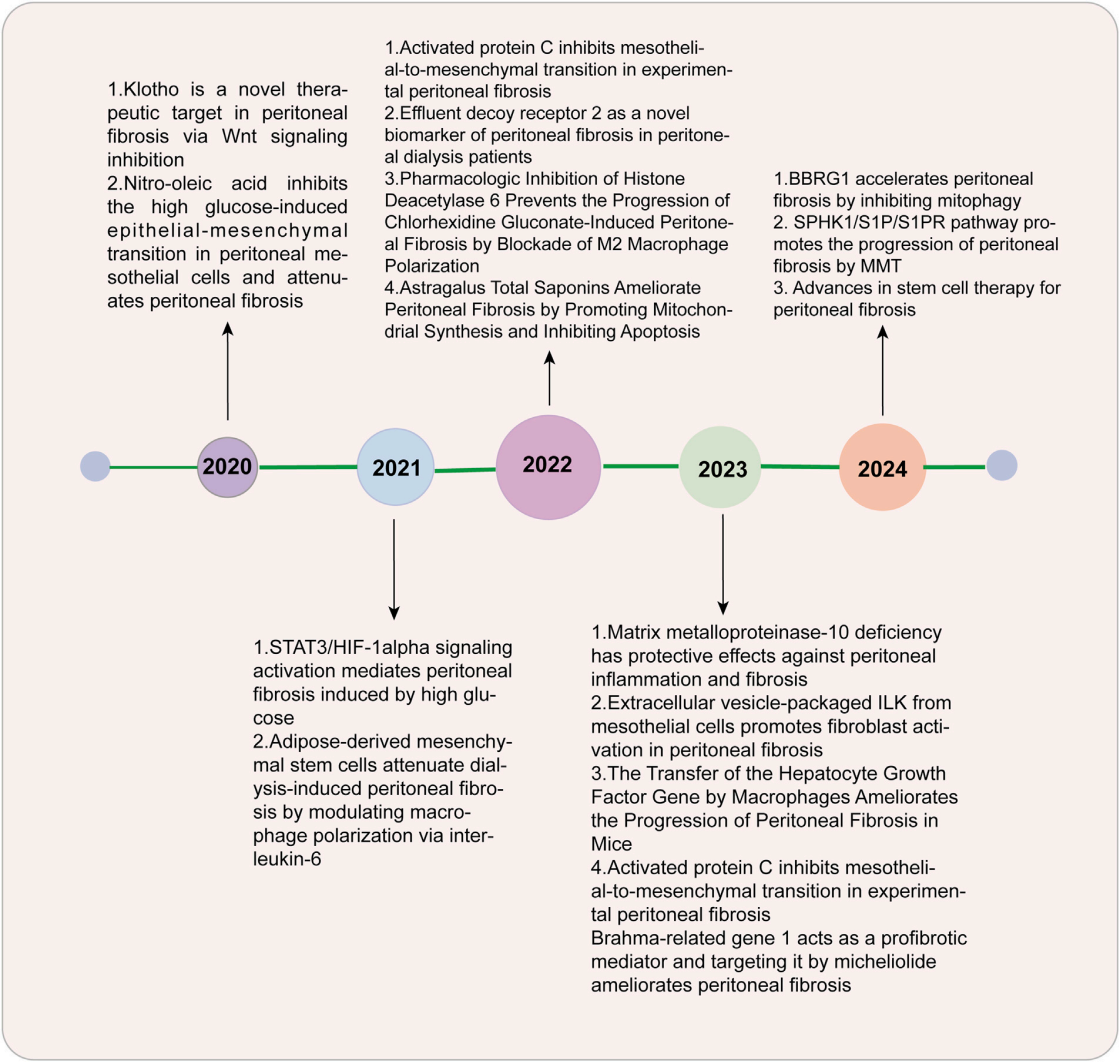
Recent research on peritoneal fibrosis.

An increasing number of patients worldwide are relying on dialysis, an alternative treatment for patients with end-stage renal disease (ESRD) (Zhou et al., 2016). It is estimated that over 10% of these patients are expected to go through peritoneal dialysis (PD) (Zhou et al., 2016). Peritoneal dialysis has been widely used due to its convenience and high economic benefits, but the occurrence of fibrosis hinders its further development (Masola et al., 2022). The peritoneal membrane (PM) is semipermeable and is used for ultrafiltration and diffusion in PD patients (Masola et al., 2022). Many vital structures are involved in mesothelial monolayers and submesothelial dense areas, such as fibroblasts, macrophages, peritoneal lymphatic vessels, and peritoneal capillaries (Branco et al., 2023a). In approximately 50%–80% of patients receiving peritoneal dialysis treatment, fibrosis may be monitored in the first 1–2 years (Zhao et al., 2023a). In fibrosis development, mesothelial cells (MCs) go through the process named mesothelial-to-mesenchymal transition (MMT) and transform into fibroblasts, which can lead to peritoneal fibrosis (PF) through the excessive production of extracellular matrix (ECM) deposited mainly in submesothelial areas (Branco et al., 2023a; Zhao et al., 2023a; Krediet, 2018). Changes in the morphology and function of the peritoneum occurred during long-term peritoneal dialysis. Therefore, PF can damage the ultrafiltration function of the peritoneum, leading to the failure of filtering excess water and metabolic waste (Krediet, 2018; Branco et al., 2023b). There are three main characteristics in PF, including the thickening of the submesothelial layer, lack of MCs, and angiogenesis (Zhao et al., 2024; Fan et al., 2008; García-López et al., 2012; Krediet et al., 2000). PF is a main risk factor for PD patients who ultimately withdraw and transfer to hemodialysis (Branco et al., 2023a). The key process of PF is MMT. The function and structure of mesothelial cells are altered due to the presence of bioincompatible peritoneal dialysate, such as glucose and glycation end products (Fan et al., 2008; García-López et al., 2012), but the mechanisms underlying these processes are still largely unclear. In recent years, more research has been conducted on PF (Figure 1), and some signaling pathways related to PF have been explored and discovered. Medications targeting these mechanisms have been validated in animal models and *in vitro* experiments, there is a hope that they will be applied for clinical practice in future.

## Pathophysiology of peritoneal fibrosis

The decrease in peritoneal filtration function can be attributed to the bioincompatibility of peritoneal dialysis fluid and the accumulation of metabolic toxins in ESRD patients (Masola et al., 2022). There are three main parts during the development of the PF: MMT, angiogenesis and inflammation (Krediet et al., 2000; de Lima et al., 2013). Peritoneal inflammation is promoted by infection and the biological incompatibility of dialysates. The definition of MMT included not only the deprivation of MCs, and deposition of extracellular matrix in submesothelial zones but also the outcome of transformation from mesothelial cells into fibroblastoid cells (Yáñez-Mó et al., 2003). Structural changes in the peritoneum, including a decrease in mesothelial cells, an increase in fibers under the mesothelium, and neovascularization, are pathological results of inflammatory injury repair and reconstruction (Selgas et al., 2006). The extracellular matrix is deposited in submesothelial areas and produced by transformed MCs, leading to PF (Li et al., 2022a; Strippoli et al., 2016; López-Cabrera, 2014). Inflammation also induces neoangiogenesis, which increases the solute diffusion surface area and PF but also reduces water permeability (Aroeira et al., 2007; Aroeira et al., 2005). In PD dialysate, glucose and glucose degradation products are the predominant components responsible for changing MC function and structure. Transforming growth factor-β (TGF-β) and vascular endothelial growth factor (VEGF) are generated by MCs and immune cells (Terri et al., 2021; Aguilera et al., 2005). Furthermore, the MMT of peritoneal mesothelial cells altered solute transport and is associated with angiogenic process (Li et al., 2023a). All these factors jointly interact and lead to the progression of PF.

Angiogenesis and fibrosis, such as the inflammatory response and MMT process, seem to be closely related (Aguilera et al., 2005; Li et al., 2023a). The reduction in MCs and PF in PD patients can be attributed to the exposure to bioincompatible dialysates or peritonitis caused by various pathogenic microorganisms. The peritoneal immune response involves different cells, such as MC and macrophages, which further mobilize different inflammatory cells. During this process, MCs and those inflammatory cells can produce abundant inflammatory mediators to establish complex interactions, resulting in inflammation, further leading to changes in the structure and function of the peritoneum. Many of these inflammatory mediators play important roles in PF, possibly by stimulating fibroblast proliferation and inducing the MMT process, leading to increased ECM deposition and further increasing the severity of PF. The level of intraperitoneal interleukin-6 (IL-6) increases due to high glucose dialysates, which causes the subsequent development of PF (Yang et al., 2020). Chemokines can stimulate neutrophils from the bone marrow and promote their development. For example, chemokine ligand 5 (CCL5), which is synthesized by peritoneal fibroblasts, can attract mononuclear leukocytes for linking (Kawka et al., 2014). Inflammation is caused by MC lesions, and the aggregation of macrophages simultaneously exacerbates this process (Zhou et al., 2016). Myofibroblasts are involved in multiple pathological processes (Kawka et al., 2014). The overexpression of these cytokines stimulates related immune cells to produce inflammatory responses. Activated resident fibroblasts secrete excess extracellular matrix, which plays a crucial role in PF (Kendall and Feghali-Bostwick, 2014). Myofibroblasts are not only produced by resident fibroblasts, but also by mesothelial cells and fibrocytes (Kendall and Feghali-Bostwick, 2014).

**FIGURE 2:**
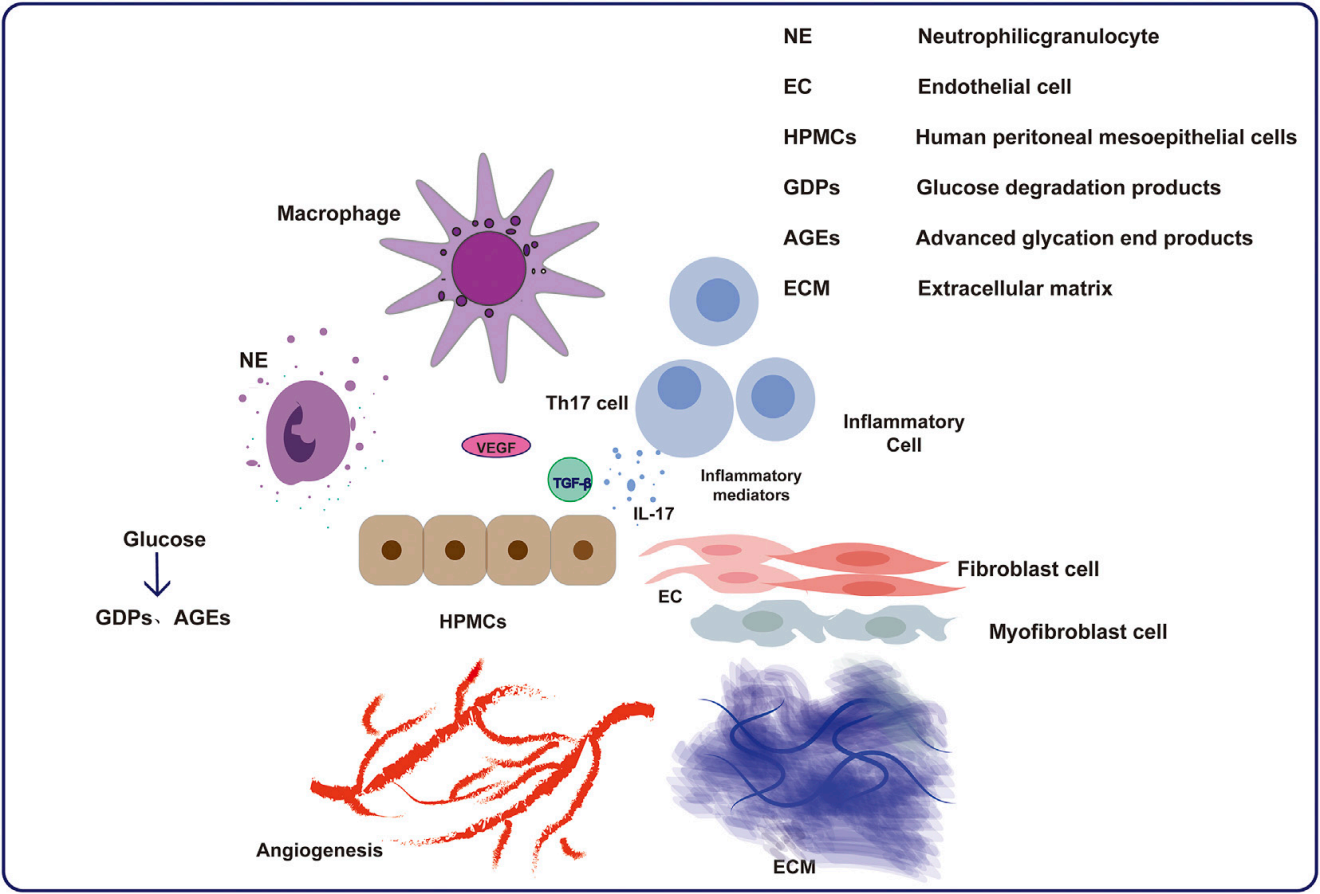
Schematic diagram of the pathophysiology of peritoneal fibrosis.

The most similar and significant changes in the peritoneum of PD patients, which means that the development of PF is related to mesothelial cell transformation and angiogenesis of peritoneal mesothelium (Apte et al., 2019). Peritoneal inflammation causes angiogenesis of the peritoneum and long-term PF in the long run. Risk factors such as peritonitis, catheterization, uremia, advanced glycosylation end products can contribute to angiogenesis. Angiogenesis plays an important role in the progression of PF, as demonstrated by the correlation between the extent of vascularization and the area of fibrotic tissues. In addition, research has shown that interleukin-8 (IL-8), fibroblast growth factor 2 (FGF-2), and especially VEGF may lead to an increase in the number of peritoneal capillaries and may further increase vascular permeability (Simons et al., 2016), causing ultrafiltration failure. VEGF plays a dominant role in mediating the functions of ECs, such as their formation, migration, and interactions. The concentration of VEGF in PD patients’ effluent increases with PD duration. When patients switched from dialysate to glucose-free PDF, the level of VEGF decreased at the same time, indicating potential relevance (Branco et al., 2023a). As shown in Figure 2, understanding the mechanism of PF and its interaction with angiogenesis is crucial for preserving peritoneal ultrafiltration function and maintaining dialysis.

## Signaling pathway of peritoneal fibrosis

The key fibrogenic factors trigger the downstream intracellular signaling pathways by interacting with their relevant receptors (Zhou et al., 2016). The main mechanism includes MMT, angiogenesis, and activation of inflammation.

The system involved in the MMT is TGF-β, and the high production of VEGF is an obvious consequence (Shirai et al., 2022). VEGF and its corresponding receptors have recently been detailed described (Zhou et al., 2016). A high level of VEGF can cause vasodilation, accelerating solute transport and decreasing peritoneal transport, ultimately causing fibrosis (Zhou et al., 2016). Glucose degradation products (GDPs) can be produced in high-glucose dialysate (Boulanger et al., 2004). Moreover, the production of advanced glycation end products (AGEs) increased either, which may cause peritoneal inflammation and even fibrosis (Boulanger et al., 2004; Khan, 2023; Cho et al., 2014). The products can motivate peritoneal macrophages, further stimulating the synthesis of cytokines and interleukins by peritoneal mesothelial cells (Kitamura et al., 2012). Toll-like receptor ligand-mediated signaling pathways and the NOD-like receptor protein 3/IL-1β signaling pathway are the main mechanisms mediating inflammation in MMT (Zhang et al., 2017).

The TGF-β superfamily regulates cell growth and differentiation. These proteins play an important role in various physiological and pathological processes, including cell proliferation, differentiation, adhesion, migration, and regulation of immune responses. TGF-β is widely involved in the formation of fibrosis either (Zhou et al., 2016). In the development process of PF, the activation of TGF-β1 is an early marker of its pathogenesis. As the peritoneum remains in biocompatible PD fluid for a long time, glucose and glucose degradation products further promote PF (Zhang et al., 2017). The activation of TGF-β is complex, including two main ways: independent and dependent on Smads (Li et al., 2018). However, Smad2 and Smad3 have distinct effects on PF. Fibrosis and dysfunction of the peritoneum are aggravated when the Smad2 gene is knocked-out, while Smad3 gene deletion prevents PF (Patel et al., 2010), which is a critical blocking target. These findings suggest that Smad3 can exacerbate PF, while Smad2 has a protective effect (Duan et al., 2014; Sun et al., 2023). Many studies also have validated that the overexpression of Smad7 can prevent and reverse fibrosis (Nie et al., 2007). Peritoneal angiogenesis can be alleviated by the Smad7 gene, which reduces capillary vessel density and inhibits the production of VEGF (Zhou et al., 2016), decreasing the activation of p38 and nuclear factor-κB (NF-κB), indicating its powerful role in inhibiting neovascularization in the PF (Silva et al., 2019).

In some situations, the entire mechanism of PF cannot be explained. The most famous Smad-independent signaling pathways related to PF have also been widely studied (Lupinacci et al., 2019). Results found that TGF-β1 causes peritoneal injury not only through Smad but also through Smad-independent pathways. Numerous studies have also illustrated the link between multiple signaling pathways in peritoneal MCs and animal models, such as the phosphatidylinositol-4,5-bisphosphate 3-kinase (PI3K), c-Jun N-terminal kinase (JNK), and TGF-β/Smad3 pathways (Liu et al., 2012). TGF-β1 activates kinase 1 (TAK1) induced by TGF- β1 regulates the transcription of target genes and plays a vital role in TGF-β1 mediated peritoneal fibrosis, by activating JNK and p38 MAPK in the Smad independent signaling pathway (Zhao et al., 2019). The Smad and Smad-independent pathways are worthy of further exploration because they are both critical for MMT development (Mo et al., 2023). Notch and heat shock proteins also act as fibrogenic or antifibrogenic factors that participate in the process of PF. In mouse models of PF, the Notch signaling pathway is highly activated (Zhu et al., 2010), increasing the expression levels of Jagged-1 and Enhancer of split homolog-1 (HES-1). Heat shock protein 70 can protect rat peritoneal MCs from high sugar PD dialysate-induced PF through the extracellular signal-regulated kinase (ERK) and TGF-β/Smad pathways (Yang et al., 2015).

Angiogenesis is characterized in PD patients receiving long-term treatment, and its degree is also related to PF. VEGF is a large gene family with significant structural and functional similarities, including not only VEGFA, but also VEGFB, VEGFC, and VEGFD (Shibuya, 2011). VEGF plays a major role in the angiogenesis of the peritoneum (Shibuya, 2011; Zhu et al., 2021). VEGF production is correlated with bioincompatible PD dialysate, growth factors, and inflammatory cytokines (Shibuya, 2011).

**FIGURE 3:**
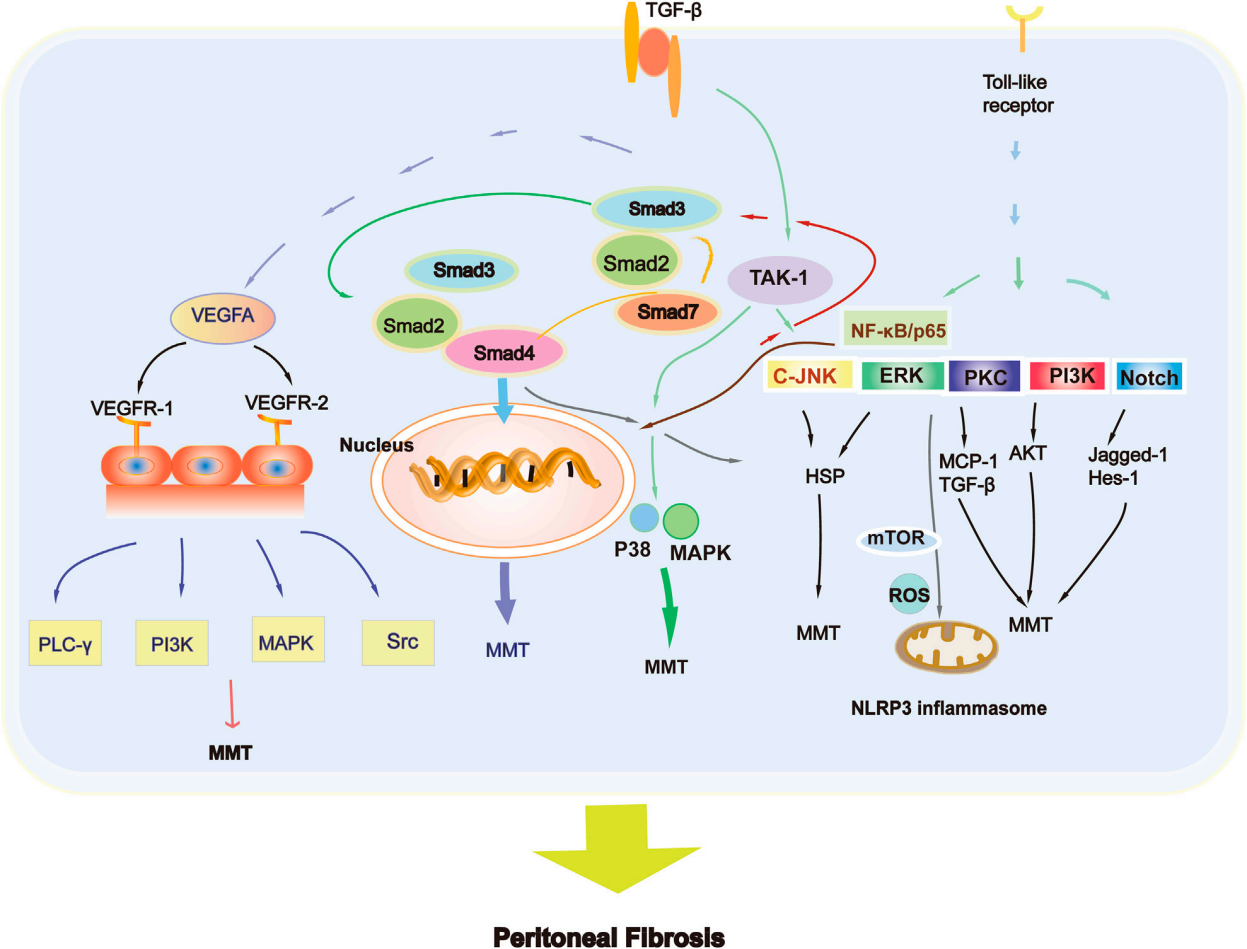
Signaling pathways of MMT in peritoneal fibrosis.

The VEGFR-1 is associated with the production of vascular endothelial growth factors, while VEGFR-2 mediates the proliferation, migration and angiogenesis of ECs (Yang et al., 2015). VEGFA triggers the process of phosphorylation of Phospholipase C gamma (PLC-γ), PI3K, mitogen-activated protein kinase (MAPK), and the Src Kinase family when binding to VEGFR-2. VEGFC and VEGFD regulate angiogenesis mostly in lymphatic ECs by binding to VEGFR-3 (Simons et al., 2016). Based on these results, the Figure 3 depicts the main MMT signaling pathway involved in PF.

## Therapeutic strategies against peritoneal fibrosis

### Based on the MMT mechanism

TGF-β1 is well known for inducing the process of MMT and PF, and thrombospondin-1 (TSP-1) can activate TGF-β1 *in vivo* experiments and induce MMT via the TGF-β1/Smad3 signaling pathway (Shi et al., 2020; Jiang et al., 2020; Heo et al., 2021). Therefore, blocking the MMT process will become an effective method to inhibit PF.

A recent study revealed that Canagliflozin could significantly ameliorate the hypoxia in human peritoneal mesothelial cells (HPMCs) (Wang et al., 2023), decrease hypoxia-inducible factor 1 alpha (HIF-1α) abundance, and ameliorate PF. These results could provide a new direction for clinical application. Empagliflozin has a clear effect on PD-induced fibrosis by inhibiting the TGF-β/Smad signaling pathway. Applying Empagliflozin treatment or downregulation of SGLT-2 expression greatly improved hypoxia-related pathological alterations of peritoneum (Shentu et al., 2021; Shi et al., 2022a). Endoglin suppresses PF by modulating the activation of the TGF-β/ALK/Smad signaling pathway (Huang et al., 2022). Inhibiting endoglin can improve PF, which will provide a new potential therapeutic for PF. Elevated dipeptidyl peptidase IV inhibitors (DPP4) activity is significantly associated with peritoneal dysfunction, and inhibiting DPP4 can protect PD patients from the PD failure (Li et al., 2021). A reduction in Nestin reportedly helps to relieve HIF1-α-induced PF, which indicates a novel mechanism of PF (Shentu et al., 2020).

Under high-glucose dialysate conditions, the expression of glucose transporter protein in the peritoneum increases, all of which are inhibited by Canagliflozin (Wang et al., 2023), a sodium-glucose cotransporter type 2 (SGLT2) inhibitor. Glucose absorption causes pseudohypoxia, and then myofibroblasts are induced by intracellular hypoxia, which leads to the upregulated expression of the glucose transporter 1 (GLUT-1), further increasing the production of profibrotic and angiogenetic factors (Krediet, 2021). The level of GLUT-1 secreted by myofibroblasts leads to a reduction in the osmotic gradient for ultrafiltration, ultimately leading to decreased peritoneal filtration function (Krediet, 2021; Feng et al., 2022).

Research has revealed a novel mechanism by which STAT3/HIF-1α signal transduction is associated with PF (Song et al., 2024). They demonstrated for the first time that inhibiting the transmission of STAT3 weakened high glucose mediated MMT and PF (Yang et al., 2021a). Peritoneal dialysis (PD) remains limited due to the destruction of ultrafiltration barriers caused by PF. In addition to traditional signaling pathways, there is another pathway involved. According to reports, an estrogen receptor 1 (ESR1) inhibitor, tamoxifen (TAM), affects fibrosis by improving the MMT of HPMC and increasing ultrafiltration rate (Zhao et al., 2023b). ESR1 significantly increases after long-term exposure to PD dialysate, TAM can reduce H19 levels by decreasing ESR1 transcription of H19 and suppressing the VEGFA (Zhao et al., 2023b). Thus, targeting ESR1/H19/VEGFA pathway indicating its prospective application in improving MMT.

Research has shown that overexpressed microRNA-26a and microRNA-200a could alleviate PF, while the decreased expression of microRNA-21a can reduce fibrosis (Si et al., 2019). These results showed that miRNAs could be potential treatment innovations (Wu et al., 2022). Activated protein C can inhibit PF, decreases the level of inflammatory mediators, reduces collagen deposition, and inhibits the process of MMT transition via the TGF-β1 and Smad3 pathways (Giri et al., 2023). Another research found that Peptide Hormones ELA and Apelin (APLN) have potential therapeutic effects on PF by suppressing MMT process (Xie et al., 2022).

### Based on angiogenesis

Angiogenesis is also an important part of the development of PF related to peritoneal dialysis (Tawada et al., 2021). In peritoneal mesothelial cells, H19 transcribed by ESR1 binds to the transcription cofactor p300, further activating the VEGFA (Zhao et al., 2023b). Targeting the ESR1/H19/VEGFA pathway provides new treatment directions for long-term PD patients (Zhao et al., 2023b). Studies showed a significant increase in the expression of the enhancer of zest homolog 2 (EZH2) in the peritoneum, which was associated with high expression of vascular markers, suggesting a link with peritoneal angiogenesis. *In vitro* studies have demonstrated that inhibition of EZH2 by 3-DZNeP or EZH2 siRNA prevents peritoneal angiogenesis via two pathways (Shi et al., 2022b): the Wnt1/β-catenin pathway and the IL-6/STAT3 pathway. Furthermore, VEGFR2/ERK1/2/HIF-1α Axis participates in *in vitro* angiogenesis, and decreased expression of EZH2 can inhibit the activation of the angiogenesis pathway.

The mesothelial cell protein αB-crystallin, which is related to angiogenesis and fibrosis, was identified. Lithium chloride (LiCl) interacts with it, which means that it can serve as a cell protective PDF supplement and may provide a translatable therapeutic strategy to inhibit PF (Herzog et al., 2021).

### Based on inflammation

Silymarin (SM) is a polyphenolic flavonoid, that is isolated from the milk thistle (Bai et al., 2023). It has a diverse pharmacological effects, such as antioxidant, anti-inflammatory, antiviral, and antifibrotic effects (Bai et al., 2023). SM can mitigate peritoneal dysfunction, and reduce the expression of fibrotic factors. The expression level of Smad7 increased, while the expression levels of TGF-β1, p-Smad2 and p-Smad3 decreased. SM may be an efficient and novel therapy for preventing the development of PF. Research also finds that selective inhibitor of type 5 of the PDE enzyme and 5-HT2B receptor may have therapeutic potential in suppressing PF by reducing inflammatory mediators (Chaturvedi et al., 2024).

Cell motility protein 1 (ELMO1) is a regulatory factor activated by Rac that regulates neutrophil chemotaxis, which suggests that the inhibition of ELMO1 could be a may be an effective target for treating peritoneal inflammation and fibrosis (Yu et al., 2023a). It has been found that PF can be alleviated by molecular hydrogen, which is conducive to eliminating intracellular ROS and inhibiting the PTEN/AKT/mTOR signaling pathway (Lu et al., 2020). Molecule hydrogen may be a may be a safe and effective curative option for PF.

Research has shown that histone deacetylase 6 (HDAC6) is closely related to PF induced by high glucose peritoneal dialysate (Shi et al., 2022c)^,^ (Shi et al., 2021a). Tubstatin A (TA) can significantly inhibit the development of PF by inhibiting HDAC6, so HDAC6 may be an innovative target for treating PF (Shi et al., 2022c; Shi et al., 2021a). Blocking HDAC6 can selectively inhibit the polarization of M2 macrophages through several key signaling pathways (Margetts, 2023). Additionally, MMPs (matrix metalloproteinase) have been reported in the context of peritoneal injury, and MMP-10 is associated with PF. Research has shown that the expression of MMP-10 is significantly increased in a mouse model of PF (Margetts, 2023; Ishimura et al., 2023). The inflammatory responses induced by the inhibition of HDAC6 significantly decreased the expression of MMP-2 and MMP-9 so it could be a potential treatment target for PF (Bontempi et al., 2022). These results indicate that histone deacetylase (HDAC) drug inhibitors may be a promising agent for treating fibrotic diseases and cancer. HDAC1-3 inhibitors induce the expression of TGFBRI mRNA-targeting miRNAs (Bontempi et al., 2022). The underlying mechanism may be summarized into HDAC1-WT1-miR-769-5p, and miR-769-5p silencing further increased the level of mesenchymal gene expression (Bontempi et al., 2022). Because HDAC1 inhibition relieves fibrosis, it may have a potential therapeutic effect aimed at PF. Results showed that miR-122-5p overexpression can cause PF by acting upon Smad5 via the Wnt/β-catenin/pathway (Liu et al., 2022a).

E-type prostaglandin receptor 4 (EP4) is significantly overexpressed in the PD patients, and researches have suggested that EP4 antagonists can alleviate the progression of PF (Luo et al., 2022). In addition, ONO-AE3-208, an EP4 receptor antagonist suppressed PF by weakening the NLR family pyrin domain containing 3 (NLRP3) inflammasome and increasing the phosphorylation of NF-κB (p-p65) (Babaev et al., 2008). Parthenolide (PTL) is an accepted inhibitor extracted from Tanacetum balsamita that can be inhibited by the NF- κB/TGF-β/Smad signaling axis, inhibits inflammation and reduces PF (Zhang et al., 2022). Chronic inflammation including peritonitis, can lead to PF, so inhibiting the activation of inflammasomes can become a therapeutic target (Zhao et al., 2023c; Arangia et al., 2023; Kadoya et al., 2023). A study has also found that a new type of antiplatelet drug has the effect of improving the inflammatory environment of the peritoneum and can alleviate PF (Liu et al., 2023).

Fatty acid oxidation (FAO) also plays a part in peritoneal fibrogenesis. Treatment of PD mice with the carnitine palmitoyltransferase 1A (CPT1A) activator C75 induces therapeutic benefits, while inhibition of FAO can lead to more severe fibrosis in PD mice (Su et al., 2023). These results demonstrated a latent therapeutic effect of inhibiting FAO. Apolipoprotein A-I (apoA-I) is the principal component of high-density lipoprotein (HDL) and has anti-inflammatory and antioxidant properties. ApoA-I and its peptide mimetics can regulate oxidative stress and the inflammatory response, reducing PF caused by peritoneal dialysis (Lu et al., 2023). Furthermore, study has also found that peritoneal dialysis increases lipid deposition in HPMC, while angiotensin II type 1 receptor (AT2) improves lipid metabolism and reduces PF by inhibiting oxidized-LDL receptor-1 (LOX-1) (Liu et al., 2022b).

HG stimulation leads to further renin-angiotensin system (RAS) activation, ultimately leading to PF. Researchers have shown that RAS-mediated ECM production is associated with lipid accumulation in HPMCs and plays a role in the low-density lipoprotein receptor (LDLr) pathway (Liu et al., 2021a). New finding suggests that the activation of free fatty acid receptor 4 could alleviate PF, which focuses on the MMT process (Zhang et al., 2024).

*Lactobacillus* casei Zhang (LCZ) has beneficial effects such as anti-inflammatory and antioxidative effects. One study revealed that it can modulate the gut microbiota, and ameliorate PF through the butyrate/PPAR-γ/NF-κB pathway, which is beneficial for preventing PD-induced PF (Wu et al., 2023).

Th17-mediated inflammation is a key element in PF. The underlying mechanism is the development of fibrosis accompanied by a slight decrease in regulatory T cells (Tregs), a kind of anti-inflammatory T cell (Raby et al., 2018). These T cells can regulate the number of inflammatory Th17 cells, which are found be involved in the development of PF. The data showed that their balance is regulated by the leukocyte antigen CD69 (Liappas et al., 2016).

Researchers have discovered Salvia miltiorrhiza and its active ingredients salvianolic acid A (Sal A) can reduce oxidative damage, alleviate peritoneal tissue inflammation and neovascularization by activating Nuclear Respiratory Factor 2 (NRF2) (Zhou et al., 2022). Many Chinese herbal ingredients have strong anti-inflammatory and antioxidant properties, which can be explored for application in PF.

### Based on apoptosis

The intrinsic antifibrotic mechanism has rarely been explored. JNK-related leucine zipper protein (JLP) has recently been found to have an antagonistic effect on TGF-β induced fibrosis process (Tian et al., 2022). JLP deficiency exacerbates PF in mice models. Knocking down JLP leads to an increased profibrotic response of human peritoneal mesothelial cell line (HMrSV5) cells to high-glucose peritoneal dialysis solution (HGPDS) stimulation, which is associated with epithelial mesenchymal transition, increased autophagy, cell apoptosis, and enhanced TGF-β1/Smad signal activation. These findings provide a new direction for novel therapeutics for PF. As mentioned earlier, autophagy may participate in the pathological mechanism of PF (Shi et al., 2021b). A recent study suggests that inhibiting the mTOR signaling pathway can activate autophagy during PD and inhibit PF (Jia et al., 2022). These results indicate that autophagy may be a potential method for preventing and treating PF.

Astragalus as a traditional Chinese medicine, which has been found to have significant anti-fibrotic effects and can be used for PF (Gong et al., 2022). One study suggested that ATS treatment reduces the thickness of peritoneal tissue in PF mouse models and increases the survival ability of peritoneal mesothelial cells (PMCs) (Li et al., 2022b). Therefore, it can inhibit PF through Peroxisome proliferator-activated receptor gamma coactivator-1 (PGC-1) mediated cell apoptosis and is an effective therapeutic agent (Ruan et al., 2024). Mesothelial cell pyroptosis stimulates downstream inflammatory responses via caspase-3 and Gasdermin E (GSDME), to activate macrophages additionally (Ruan et al., 2024). GSDME deficient mice are immune to PD induced PF and ultrafiltration failure (Ruan et al., 2024). Therefore, melatonin can alleviate mesothelial cell pyroptosis and reduce PF.

## Other novel therapeutic strategies

### Stem cells

Research has shown that adipose derived mesenchymal stem cells (ADSCs) have immunomodulatory and antifibrotic effects on PF (Yang et al., 2021b). *In vitro* experiments have also shown that mesenchymal stem cells have a positive effect on improving PF and can serve as one of the targets (Yu et al., 2023b; Nagasaki et al., 2021). In PD-related PF, mesenchymal stem cells are in an inflammatory filled state, such as TGF-β1, to polarize macrophages into M2 phenotype by secreting IL-6 (Shao et al., 2023; Zhou et al., 2023).

### Gene therapy

Brahma related gene 1 (BRG1) is a key factor in organ fibrosis, and micheliolide (MCL) has been found the ability to inhibit PF in mice (Li et al., 2023b). A recent study revealed that BRG1 may be a mediator of PF and MCL targeting the asparagine (N1540) residue of BRG1 may be a new therapeutic strategy for PF (Li et al., 2023b).

Hepatocyte growth factor (HGF) is a classical gene that plays a part in antifibrotic role (Obata et al., 2023). Research has shown that sonoporation-based hHGF transfection plays a significant role in early PF (Nishimura et al., 2021). Moreover, the transplantation of HGF-M can inhibit the development of PF and may have a potential effect on alleviating PF (Yoshimine et al., 2021).

### MicroRNAs (miRNAs)

MiRNAs have been shown to be associated with various diseases and have the potential to serve as disease biomarkers and therapeutic targets (Brown and Naldini, 2009). Restoration of miR-15a-5p restrained the inflammation and fibrosis of HPMCs, and the miR‐15a‐5p/VEGFA pathway may be potential targets for preventing PF (Shang et al., 2019). MiR-199a-5p and miR-214-3p play important role in PF by targeting claudin-2 and E-cadherin. Overexpression of miR-30a can reduce the increase of Snai1 induced by TGF-β1 and inhibit the occurrence of MMT (Che et al., 2017). miR-129-5p pathway has significant roles in EMT via targeting SIP-1 and SOX4 by inhibiting EMT process (Xiao et al., 2015). miR-30b, miR-145 and miR-200 family are all involved in the occurrence of MMT (Liu et al., 2014; Wu et al., 2019; Guo et al., 2018; Chu et al., 2019). These findings illustrated that numerous miRNAs are involved in PF that they may be served as novel therapeutic targets for PF.

## Remaining drugs and therapeutic mechanisms

Nintedanib, a multiple tyrosine kinase inhibitor, can inhibit MMT and attenuate PF (Liu et al., 2021b). It also has an effect on reducing inflammation and angiogenesis. It has therapeutic ability in the prevention and treatment of PF (Cui et al., 2022).

Saikosaponin D (SSD), a monomeric substance extracted from the Bupleurum chinense, has been discovered to slow down PF and have anti-inflammatory and anti-fibrotic effects (Ruiqi et al., 2021). The silent information regulator sirtuin 1 (SIRT1) ameliorated PF via TGF-β signaling by inhibiting the expression of protein matrix in both *in vivo* and *in vitro* experiments (Guo et al., 2021).

Peritoneal EVs regulate the mutation of mesothelial cells and fibroblasts in PF (Szebeni et al., 2024). EVs produced by mesothelial cells are rich in integrin-linked kinase (ILK), which can activate fibroblasts via the p38-mitogen-activated protein kinase (MAPK) signaling pathway (Huang et al., 2023a). Extracellular vesicles also have an impact on the process of PF by acting on TGF-β (Szebeni et al., 2024; Huang et al., 2023a). In the future, targeting EVs or ILK may provide new therapeutic directions for PF.

1,25-dihydroxyvitamin D3 [1,25- (OH) 2D3], also known as active vitamin D3, is involved in various physiological metabolic process in the body (Da et al., 2020). *In vitro* and *in vivo* experiments revealed that the progression of PF can be delayed by regulating the expression levels of heat shock protein 47 (HSP47) and connective tissue growth factor (CTGF) (Da et al., 2020). Therefore, these results demonstrated that blockade of 1,25-(OH)_2_D_3_ can ameliorate peritoneal thickness and has a good effect on PF.

Simultaneously changing the composition of dialysis fluid may ameliorate PF, and lactate-based peritoneal dialysate is implicated in the development of peritoneal structural and functional changes (Masola et al., 2021). The study confirmed that steviol glycosides (SG) exhibit better biocompatibility as a penetrating agent than glucose (Kopytina et al., 2022). Therefore, changing the gradient of the dialysate, and using more biocompatible osmotic agents will be the direction for preventing PF (Pap et al., 2023). Biocompatible low glucose degradation products have shown superiority compared to traditional PD dialysate (Elphick et al., 2018; Moinuddin et al., 2024). Table 1 roughly summarizes the treatment strategies for improving peritoneal fibrosis based on different mechanisms in current research.

**TABLE 1:** The brief overview of therapeutic strategies in peritoneal fibrosis.

Related mechanism	Treatment strategy	Signaling pathway
MMT	Canagliflozin	HIF-1α
	Empagliflozin	TGF-β/Smad
	Endoglin	TGF-β/ALK/Smad
	DPP4 inhibitor	TGFβ/SMAD3
	Nestin	HIF1-α
	Tamoxifen	ESR1/H19/VEGFA
	Protein C activation	TGF-β1/Smad3
	Peptide Hormones ELA and Apelin (APLN)	TGF-β/Smad
Angiogenesis	Tamoxifen	ESR1/H19/VEGFA
	3-DZNeP/EZH2	Wnt1/β-catenin
	siRNA	IL-6/STAT3
	EZH2 depressor	VEGFR2/ERK1/2/HIF-1α
	Lithium chloride (LiCl)	αB-crystallin/TGF-β1
Inflammation	Silymarin (SM)	TGF-β/Smad
	PDE enzyme and 5-HT2B receptor	5-HT/TGF-β1
	ELMO1	PI3K and mTOR
	Molecular hydrogen	PTEN/AKT/mTOR
		HDAC1-WT1-miR-769-5p pathway
	Histone deacetylase 6 (HDAC6)	IL-6/STAT3
		Wnt1/β-catenin
	Tubstatin A (TA)	Nuclear factor kappa B (NF-κB) (p-p65)
	EP4 antagonists	(NF-κB) (p-p65)
	Parthenolide (PTL)	NF-κB/TGF-β-Smad
	Indobufen	NF-κB/NLRP3
	CPT1A activator (C75)	TGF-β1/Smad3
	Angiotensin II type 1 receptor (AT2)	AT2- LOX-1
	Free fatty acid receptor 4 activator	FFAR4/CaMKKβ/AMPK/mTOR
	*Lactobacillus* casei Zhang (LCZ)	butyrate/PPAR-γ/NF-κB
	Salvia miltiorrhiza	Nrf2 and NF-κB
Apoptosis	JNK-related leucine zipper protein (JLP)	TGF-β1/Smad
	Astragalus	PGC-1 mediated cell apoptosis
	Melatonin	p38-MAPK and NF-kB
Others	Stem cell	adipose derived mesenchymal stem cells
	Gene therapy	Brahma related gene 1 (BRG1)
		Micheliolide (MCL)
		Hepatocyte growth factor (HGF)
	MicroRNAs	TGF-β1
		SIP-1 and SOX4

## Conclusion and future exploration

Fibrosis is presented as increased fiber tissue and reduced mesothelial cells, which can occur in any tissue or organ and can lead to organ destruction and malfunction (Lai et al., 2024). PF has long been the main complication in PD patients, leading to the failure of peritoneal dialysis and increasing the burden of medical expenses. Just as renal fibrosis involves multiple mechanisms (Huang et al., 2023b), there is an urgent need to discover more biomarkers for early identification and intervention in PF. Peritoneal inflammation, endothelial mesothelium transformation, and angiogenesis are the three main mechanisms of PF. Extensive researches have been conducted on the mechanisms of MMT, inflammation, angiogenesis and apoptosis, which are closely associated with the process of PF. Numerous studies have also illustrated the new development of PF in MicroRNAs, gene therapy and stem cells. Many targeted drugs and new treatment methods have been found to produce marked effects via different mechanisms (Hu et al., 2023). Some medications have also been applied in clinical practice. In addition, upgrading the component of peritoneal dialysis fluid will also be a promising option for improving PF. Although further trials are needed, the current results illustrate that there will be additional novel approaches for the treatment of PF, that can maintain peritoneal function and improve the current status of peritoneal dialysis treatment.
